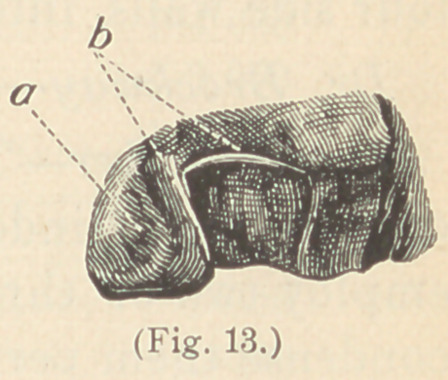# The Herbst Method of Filling Teeth

**Published:** 1885-08

**Authors:** C. F. W. Bodecker

**Affiliations:** New York


### Independent Practitioner.

Vol. VI. August, 1885. No. 8.

### Original Communications.

### THE HERBST METHOD OF FILLING TEETH.

BY C. F. W. BODECKER, D. D. S., M. D. S., NEW YORK.

AN ADDRESS DELIVERED BEFORE THE DENTAL SOCIETY OF THE STATE OF NEW YORK, MAY 13TH, 1885.

*Mr. President and Gentlemen :*

I have no doubt that you have heard or read of the new process of introducing filling materials, such as gold, tin and amalgam, into cavities in human teeth by means of engine-burnishers. It is some years since Dr. Shumway, of Plymouth, Mass., advocated rubbing gold into cavities by the use of ivory points, but he did not employ the dental engine. I have been informed that Dr. Bronson, of New York, had more recently used ordinary engine-burnishers for the introduction of amalgam only.

About six years ago Wilhelm Herbst, of Bremen, Germany, who is not conversant with our professional literature, conceived the idea of introducing gold into cavities by means of smooth-faced burnishers. Herbst's discoveries were, therefore, made independent of Shumway and Bronson. The method now practiced is as follows: The gold is introduced into the cavity in the form of pellets, or cylinders, which are burnished down against the walls of the cavity, beginning with a hand instrument and continuing with a roof-shaped point in the engine, with which the gold is condensed thoroughly into every depression and corner of the cavity.

The advantages claimed for this method are :

1st. Better adaptation to the walls of the cavity than it is possible to obtain by any other system.

2d. The saving of about one-half the time required for other methods.

3d. Some of the most difficult operations (as proximate surfaces of the molars and bicuspids) by this method are very easily performed.

4th. Gold can be perfectly adapted to the thin walls of enamel without danger of fracture.

5th. The introduction of gold, when done by this method, is much less annoying to the patient, and less laborious to the operator.

The instruments used for this method are mostly ordinary smooth burnishers, of which there are three sets; one set of engine points, one

1 2 3 4 5 6 7 8 9 10 11 12 13 14 15

(Fig. 1.)

set of hand instruments, and one set of bent hand instruments. The two former sets were designed by Herbst, the latter by Dr. Frank Abbott. The engine set is composed of fifteen instruments, but only a few of these are very much used, although we may meet with cases in which all can be employed. The most important of them is the roof-shaped instrument No. 5, of which there should be several sizes. These can easily be made out of a broken bur, as follows: The broken instrument is put in the hand piece of the engine, which, while rotating rapidly, is ground upon an Arkansas stone. The instrument should lay obliquely upon the stone, like a pen in writing, and be quickly moved, drawing it from one side of the stone to the other.

The instruments Nos. 2, 3 and 4, since they have been replaced by hand instruments, are but seldom used for gold fillings. The larger instruments, Nos. 1, 2 and 6, are mostly intended for the use of amalgam and tin. The pointed instruments, Nos. 7 to 15, are used for finishing and condensing the edges of proximate fillings. To this set I have added three very small, round points, in shape resembling a round cavity bur. They are designed for the use of small proximate cavities in incisors, and to me have proved of great advantage. (See Fig. 10.)

Mr. R. S. Williams, of this city, has devised a roof-shaped condensing instrument with corrugations, which possesses some advantages. All these instruments should be highly tempered. When, therefore, by continual use they become soft, they should be re-tempered.

About a year ago, when I gave a clinic before the First District Dental Society, Dr. Wheeler, of Albany, handed me a burnisher made of blood-stone, with which I finished the filling. The gold will not cohere to agate or blood-stone as it will to steel, thus obviating the necessity for the frequent cleaning of them upon emery, crocus or rouge cloth. These agate and blood-stone points have lately been imported by Dr. Timme, of Hoboken, New Jersey. They may be run at a high rate of speed, without perceptibly heating the gold. Gold introduced with agate or blood-stone becomes perfectly polished, and if another layer of gold is to be added the filling must previously be roughened by a roof-shaped or Williams instrument, else the several layers of gold may become separated again.

(Fig. 2.)

(Fig. 3.)

(Fig. 4.)

The hand instruments designed by Herbst (Fig. 4) are five in number. Four are pear-shaped and one is a very fine roof-shaped instrument. Nos. 1, 2, 3, 4 are intended to condense and bring the gold to its proper place before the roof-shaped engine instruments are employed. No. 5 is an exploring instrument, which is to be pressed over the surface of the gold, especially the first layer, to discover the imperfectly condensed places.

Dr. Abbott's set (Fig. 3) is composed of bent burnishers, for those places which a straight instrument cannot perfectly reach. These instruments will condense the gold almost as perfectly as the engine burnishers, and offer special advantages for this method.

When I first read an account of this method I placed very little confidence in it, and did not give it much attention until about two years ago, when Dr. Herbst sent me a patient, that I might examine some of his operations in the mouth. These fillings were beautifully done; the edges were absolutely perfect, clean cut, and as well finished as they could be when done by the best of our mallet operators. Last year, when in Europe, I stayed with Dr. Herbst a week, experimenting with him all the time. I took from America a Bonwill mechanical mallet, that I might be sure that I had an instrument with which I was acquainted. Herbst first filled a steel matrix, somewhat resembling a bicuspid tooth, in which a moderately large hole had been drilled. In this cavity were a number of small pits, drilled at right angles to it. When filling this cavity with the mechanical mallet I was obliged to confess my inability to reach the pits with that instrument. Of course, no one would think of preparing a cavity in the manner in which the matrix was prepared, but for the sake of arriving at some definite conclusions we proceeded. I consumed about twice the time that Herbst did, and when we took out the plugs mine were imperfect, while his were perfectly polished upon the surfaces next the steel. This, of course, induced me to pursue experiments further. The plugs made by Herbst himself, by rotation, when weighed were found to be a little lighter than those I made with the mallet, but the adaptability was very much superior.

At the May clinic of the First District Dental Society, we performed some experiments, first in glass tubes which I have here for inspection. One was filled by Dr. E. P. Brown, with the Bonwill mallet, in twenty-nine minutes ( marked Brown) ; one was filled by Dr. Abbott, with hand burnishers, employing the same process as the Herbst method, save that he did not use an engine (marked A), the filling being completed in six and a half minutes, and my tube (marked B) was filled in six minutes, the gold being introduced by the Herbst method. By comparing these three tubes, you will observe that with the Herbst method can be obtained the most perfect adaptation possible, a result which cannot be reached by the mallet system. We experimented further at the clinic, with a matrix made of steel, which was furnished by Dr. Abbott. Dr. Brown, with the electro-magnetic mallet, filled that matrix in forty minutes, and his plug weighed eighteen grains. Dr. Abbott employed the rubbing method, using his hand instruments, without the engine, and it took him eight minutes, he employing some one to anneal and feed the gold. This plug weighed seventeen and a half grains. I used eleven minutes, and my plug weighed fourteen and a half grains, rubbing with the engine. But here I must state that this experiment was not quite conclusive. In the first place, my matrix was not completely filled, and the engine I had at my disposal was altogether worn out. I have since made some experiments with steel points (in the former experiment I was using agate points from the beginning to the end), and in these later experiments I have brought up the weight of the plugs, made in the same matrix in about the same time, to seventeen and three-quarter grains. On removing these fillings, Dr. Abbott's as well as mine separated into two parts, but Dr. Brown's stayed intact. The adaptation against the steel wall was absolutely perfect in my plug ; in Dr. Abbott's it was nearly perfect, but in Dr. Brown's there were several places where could be seen the layers of the foil. From these and other experiments I draw the conclusion that, against the walls of a cavity, nothing can produce so perfect an adaptation as the Herbst process. When such a filling is taken out, it presents a perfectly polished surface. Experiments that I have made since that clinic have demonstrated that steel points will condense the gold better than agate. There is more penetration to the former, and blood-stone ranks between the two. But if the steel points be continually used in the engine they become coated with a film of gold, and they must be frequently rubbed upon tin, crocus cloth, or something that will remove it.

Dr. Brockway asks if this is not because one can bear on harder with a steel point. I think not, because by using straight pressure upon a large sized agate almost as much force can be used as with a steel instrument. The small agate points are, of course, very frail. I have found that a plug made entirely with agate points does not weigh as much as when condensed by steel. But when the new hand instruments are first used to put the plug in place, and followed by the cone-shaped steel instruments, the condensation of the gold is perfect. The use of the hand instruments will, to a certain extent, save the cleaning of the engine points, thus making the agate or blood-stone unnecessary.

In experimenting with this method, Herbst, as well as myself, has tried the gold of most manufacturers, and found that almost every kind can be used, but that manufactured by Carl Wolrab, of Bremen, Germany, is particularly well adapted. There is no preparation of gold with which I have had such good success as with this, and I think that those gentlemen who have used it will corroborate me.

The forms of gold best adapted for the Herbst method are very soft cylinders, of which the larger sizes are the most useful. If foil is used, Nos. 3, 4 and 5 are the best for the purpose. The leaves are cut into halves, and rolled into a rope between the fingers or with a napkin, and cut into pellets of required length ; or the sheet may be divided into squares measuring from one-half to one inch, which, by means of a pair of foil tweezers or the fingers, are formed into pellets. The foil, as well as the cylinders, should never be annealed when used in the first layers of the cavity, except it be a contour operation.

0 1 2 3 4

(Fig. 5.)

One of the essential rules for filling by the Herbst method is the conversion of all complicated cavities (such as proximate ones), which possess but one, two or three lateral walls, into simple ones (such as cavities involving the grinding surfaces of molars, and having four lateral walls), which is accomplished by the application of a proper matrix. The matrices used for this purpose are either made of steel, Brown's polishing metal, wood or shellac, or the Jack matrices may be employed. Those used for the proximate surfaces of molars and bicuspids are the forms devised by Dr. Louis Jack, or they may be made out of a piece of thin watch-spring, wood, or the loop matrix may be employed. The watch-spring matrices are made out of a piece of watch-spring saw, such as may be obtained from any of the dental depots, in the following manner: A piece of saw, about half an inch long and as broad as the cavity is deep, is cut off and heated over a spirit flame until it is dark blue. The points of the matrix which are designed to rest on the cervical edge of the cavity ought to be well rounded off, that in cavities extending under the gum it may be pushed down without injuring either the lingual or buccal portion of the gum. The lateral ends of the matrix must be bent around the lingual and buccal portion of the tooth to be filled, like a clasp, so that the matrix when in position and viewed from the grinding surface shall present a semi-lunar form. When thus prepared it may be secured by one or two wedges or pins of wood, inserted, one from the buccal, the other from the lingual side. These wedges should be placed near the gum, between the matrix and the adjoining tooth, firmly pressing the former against the edges of the cavity. In adjusting one of these matrices care should be observed that in mesial cavities it does not quite reach the grinding surface of the tooth, or it will obstruct the entrance to the cavity. All these steel matrices may be saved and used many successive times. When two cavities in bicuspids or molars face each other, if the former plan does not answer, the matrix, after it has been placed in position, may be secured by filling one of the cavities with cotton or shellac. In some cases, where there is sufficient separation between the two teeth, Dr. Jack's matrices should be used, as they will restore the contour of the tooth better than any other. In a former publication, I mentioned that in proximate cavities of molars and bicuspids Herbst did not level the edges of a cavity very much, because he adjusted the matrix so closely that none of the gold had to be trimmed off. But I have since found that the loose adjustment is not only no objection, but an advantage. The matrix, if adjusted loosely, will permit the gold to project from the cavity somewhat, and by means of the pointed burnishers, Nos. 8 to 15 (Fig. 1), it can be rubbed over the edges, but the filling of a cavity, when the matrix is not entirely firm, requires a little more care and practice than the filling of a cavity against the walls of which the matrix is carefully secured.

When several walls of a back tooth are to be restored, or the adjoining tooth is missing, the loop matrix (Fig. 6) may be employed with advantage. The loops, as supplied by the dental depots, are much too thick for the majority of cases.

Extra loops are very easily made from a thin watch-spring saw, to fit every case where a loop matrix is applicable. If the circumference of the tooth to be filled is very much greater at its grinding surface than at the neck, the loop may be annealed and hammered as required, or the loop may be given such a form that when bent together it will be larger on one border than on the other.

(Fig. 6.)

In some instances, where the lingual walls of the upper front teeth are not broken too much, I have made use of a thin piece of Brown's polishing metal, or steel. At first I used steel, as mentioned before, in the form of a thin watch-spring saw. But I found that this material in some instances was difficult to adjust perfectly against the lingual walls of the teeth. Then I used Dr. Brown's polishing metal, which I found of great service. It is a metal very easily bent in shape, easily adjusted, and easily removed again, and it can be readily held in position with the fingers. In some instances, where the perforation in the lingual wall is extensive, I have held a small piece of thick blotting paper under the Brown metal, which will then stand all the pressure required for the introduction of the gold. To form the matrix I take a piece of the thin metal, about one inch in length and wide enough to completely cover the cavity in the lingual surface of the tooth to be filled, insert it between the proximate surfaces of the incisors containing the cavity, and bend one end of it so as to cover the cavity in the lingual surface; the other end is bent out of the way, over the labial surface of the adjoining tooth. (See Figures 11 and 12.)

(Fig. 7.)

For the proximate surfaces of the incisors, when their lingual walls are broken, as well as in contour operations, a matrix of shellac is employed, which may be made in the following manner: A piece of shellac, the size of a large walnut, is warmed over an alcohol lamp to the consistency of putty, and after the rubber dam has been adjusted this is pressed against the lingual wall, extending a little over the cutting edges of four or six of the teeth. After it has become hard it may be removed from the mouth, and if any of the shellac is pressed into the cavity it must be carefully removed by cold excavators. It is trimmed as desired, and then put back again in its place. (See Fig. 8.)

When the labial wall of such a cavity is broken to such an extent that the gold can be easily packed from the labial surface, an additional steel matrix must be applied against the proximate surface of the cavity. This matrix may be secured either by pins, wood, or cotton, or it may be warmed and pressed into the lingual surface of the shellac matrix, as in Fig. 7.

(Fig. 8.)

This steel matrix, made of a thin piece of clock or watch spring, must not quite reach to the labial surface of the tooth, as it may offer an obstruction to the introduction of the gold. Herbst says that the same matrix represented in Fig. 8, has been used in the mouth from which this cast and drawing were made.

The matrix used for contour operations of incisors is made in a similar manner, but besides the steel matrix of the proximate surfaces, an additional one should be put in for the restoration of the cutting edge of the tooth. (See Fig. 9.)

(Fig. 9.)

The preparation of cavities to be filled by the Herbst method is, with a few exceptions, the same as for any other method. In no instances are deep undercuts or starting pits necessary, but the cavity should be so shaped that it will securely hold the filling. The edges are prepared in the usual manner. In a former article (See INDEPENDENT PRACTITIONER for November, 1884) I stated that all proximate cavities of the molars and bicuspids must be made accessible from their grinding surfaces, but at present I do not prepare any cavities for this method in a diff. rent manner from that for any other system of filling.

The introduction of the gold is the principal new feature of the Herbst method, and if certain general rules are observed this becomes quite simple. It is probable that some dentists who have made a failure in their experiments with the Herbst system have done so because of their ignorance of the basal principles of the method.

During the introduction of the filling material, the gold, which (when unannealed) apparently shows no signs of cohesion, working as soft as tin foil when burnished, becomes cohesive. The reason for this I am unable to give; there is certainly not enough heat developed to cause it to become cohesive, nor should electrical action during rotation exert any material influence upon it. It is certain that Wolrab's German gold possesses this property in a very marked degree, and it is largely owing to this that the Herbst method is crowned with success.

The main rule to be observed in the starting of a filling is, that the first layer must be sufficiently large, so that when condensed it will lie securely in the cavity without being supported by an instrument. When too little gold has been put into the first layer, or when a number of too small cylinders are used, and an attempt is made to condense them, the gold will roll about under the instrument, and become too hard to be again adapted to the walls and edges of the cavity. The same condition will be observed when the first hand instrument used in condensing the gold has been too small.

The Herbst hand instruments, while pressing hard upon the gold, are rotated in the hand about one-half or three-quarters of a turn, but the Abbott instruments are merely moved from side to side. By a rotary motion the gold is much better condensed than by simple pressure. Before the hand instruments are used upon a newly added layer of gold, they should be rubbed upon a piece of No. 1 sandpaper. After the gold has been thus condensed, the perfect adaptation is obtained by the roof-shaped or conical point in the engine. This instrument is to be ground upon an Arkansas stone or a piece of sandpaper while revolving, holding it in about the position a pen is held upon the paper while writing. After it is passed over the sandpaper or Arkansas stone, and is perfectly clean, it is, while rotating, pressed upon the gold, condensing it thoroughly into every depression of the cavity. In condensing, this instrument should not be held upon one spot, but be moved around, and especially along the edges of the cavity. In using these points care should be taken that the engine is not run too fast, and that the burnisher, while in motion, is not allowed to be in contact with the gold longer than from one to three seconds, lest the gold be heated to such an extent as to cause discomfort, or even great pain to the patient. When the first layer of gold has been thoroughly condensed with the roof-shaped instruments, the hand instrument No. 5 (Fig. 1), while rotating, is pressed firmly around the edges and depressions of the cavity. If this makes any deep pits in the gold, it proves that in these places it was not perfectly condensed. and a smaller roof-shaped instrument than that used in the first instance should be employed in the engine to condense these places. All deep pits present in the first layer of gold should now be filled up with very small gold cylinders, and thoroughly condensed until the surface of the gold is even. All succeeding layers of gold are manipulated in the same manner. In some situations, as in the buccal walls of molars and bicuspids, when the gold cannot be condensed by direct action of the instrument, the right angle attachment, or an Abbott hand instrument should be employed.

When a number of layers have been secured, and all the walls and edges of the cavity are covered, it will sometimes be found necessary, if the operation is to be concluded by the Herbst system, to slightly warm or anneal the gold. In view of the results of the experiments made, I deem it safer, especially for a beginner in this system, to finish an operation in the old accustomed manner. The experiments have demonstrated that the walls of a cavity, when filled by the Herbst system, are more perfect, although the plug does not weigh quite as much as one made by the mallet. Although we know that the specific weight of a gold plug is not of great importance, yet the more solid a filling is upon the grinding surface the better it will wear.

Tin is introduced in the same manner as gold, either in the form of foil, or as Robinson's metal. Nos. 4 to 6 foil is cut in half, and is made into a rope with the fingers or a napkin, and cut into pieces of the desired length, which ought to be used when prepared, as tin oxidizes when in contact with the air for any length of time.

About six months ago Herbst sent me the preparations which I now exhibit. They are tin fillings, coated with a layer of gold. He then supposed that he had discovered something new, but you are aware that this combination of tin and gold has been in use for many years. Tin, as a filling material, has some very desirable properties. I have, therefore, of late used a thin layer of it upon the cervical portion of every proximate cavity where it is out of sight. For this the Herbst method offers special advantages, as a very thin layer of it can be burnished around the edges and walls of the cavity very perfectly, and with great ease.

I will now describe the method of filling special cavities.

I. Distal surfaces of bicuspid and molar teeth, involving the proximate and grinding surface. After the cavity has been prepared in the usual manner, and the rubber dam has been adjusted, the matrix is applied in the manner mentioned above. This being in place, and the cavity having been thoroughly disinfected and dried, everything is ready for the introduction of the gold. Two, three, or four large gold cylinders are loosely placed in the cavity with a pair of foil tweezers, then a hand instrument, either Herbst's or Abbott's, as large as the entrance of the cavity will admit, is cleaned upon fine sandpaper, and with this, while rotating, the gold is firmly compressed, first into the bottom and then against the side walls of the cavity. The gold is then thoroughly condensed, especially against the matrix and edge of the cavity, with one of the roof-shaped instruments No. 5, (Fig. 1), and, as mentioned above, examined with the thinnest hand instrument. In this manner layer after layer is introduced until the cavity is filled. If desired, the last layer may be packed by the mallet.

II. The introduction of gold into cavities involving the mesial and grinding surfaces of molars and bicuspids is a little more troublesome, although when the Abbott instruments are employed the filling of these cavities is simplified. The anterior edges and walls of the matrix in these localities cannot always be reached by direct action of the engine. A roof-shaped instrument in the right-angle attachment is therefore indispensable, which, while rotating, is firmly pressed forward against the matrix and edge of the cavity.

III. Occasionally we meet with cavities in the mesial or distal surfaces of bicuspids, near the gum, which, if the tooth structure between the cavity and grinding surface is strong enough, may be filled from the buccal surface. In these instances we use as a matrix a thin piece of clock spring, about one inch in length, and push it between the two teeth. If the cavity is situated in the distal portion of the first bicuspid, the buccal end of the steel-spring matrix is bent backwards as much as possible, around the second bicuspid, that it may not obstruct the entrance of the cavity. When the cavity is situated in the mesial portion of the second bicuspid, introduction of the gold is accomplished in the same manner as described above.

IV. The packing of gold in cavities upon the grinding surfaces of molars is somewhat different. The first layer introduced must extend over the whole surface, and be sufficiently thick to lie quietly when the instrument No. 5, (Fig. 1) is used to condense it into the several depressions of the cavity. To facilitate the packing of the succeeding layers, the gold cylinders may be warmed, or even slightly annealed. When the operation is nearly completed, I would advise, especially for beginners, that the filling be finished by the mallet.

V. Proximate surfaces of the incisors can be very easily and quickly filled by this method. The cavities are prepared in the same manner as for filling by any other system, but no deep starting points are made, a slight round undercut at the cervical wall and one towards the cutting edge of the cavity being amply sufficient. The separation required for this method is not more than when the cavity has been prepared for other methods. Herbst fills all these cavities with a No. 5 instrument, but I use a burnisher made exactly like a small round bur, which works very satisfactorily. (See Fig. 10.)

(Fig. 10.)

When there are two cavities to be filled which face each other, a matrix made of a thin piece of clock or watch spring may be used to great advantage. This matrix may be either inserted into a piece of shellac (See Fig. 7), or it may be used in the same manner as described in paragraph III. Herbst formerly filled two opposite cavities of incisor or bicuspid teeth in the following manner: When the first layer in both cavities had been thoroughly condensed, more gold cylinders were added in both cavities, and condensed as though there were but one. When sufficient gold has been introduced the two fillings are separated by a No. 15 (Fig. 1) instrument (an ordinary fine sewing needle secured in a mandrel or chuck), which, while rotating, is pressed through the median line of the fillings in several places. The two fillings are further separated by means of a thin clock-spring saw, the edges thoroughly condensed with one of the pointed burnishers Nos. 12 to 14, (Fig. 1), and finished.

(Fig. 11.)

(Fig. 12.)

VI. Proximate surfaces of incisors with broken lingual walls are comparatively easy to manipulate when a matrix is applied to the broken wall. This may be made either of shellac, a piece of thin clock spring, or Brown's metal, as described in a preceding paragraph. When the labial wall of an incisor is not broken away, the cavity may be opened and filled from the lingual surface, thus avoiding the appearance of gold on the labial surface, and the annoyance and loss of time in wedging. If the teeth stand in such close proximity that they will not admit a fine finishing strip, a small wooden wedge must be inserted near the gum, after which the cavity is prepared and filled in the usual manner. When there are two cavities facing each other to be filled, a thin clock-spring matrix should be inserted between the two cavities in the same manner as when they are to be filled from the labial surface. (See Par. V.) If a cavity is situated in the mesial surface of a right upper central incisor, the clock-spring matrix (about one inch long and wide enough to cover the cavity) is inserted between the two centrals, when the labial portion of the spring is bent over the labial surface of the right central (the tooth to be filled), while the lingual portion is bent against the lingual wall of the adjoining (left) central.

(Fig. 13.)

VII. When, however, the labial as well as the lingual walls of incisor teeth are broken to such an extent that the gold can be easily packed from the labial surface, a matrix, represented in Fig. 13, or in some instances the above-mentioned clock-spring matrix, may be employed. The gold is introduced in the usual manner.

VIII. Contour operations of the cutting edges of the front teeth have only been accomplished within a comparatively short space of time. When I last saw Herbst, I showed him a patient for whom I had inserted in the lower jaw five or six very large fillings. Two of the centrals were largely contoured, and this was the first work of the kind he had ever seen. Herbst was quite surprised when he saw these operations, and remarked: "I will not rest until I can produce the same operation with my method." Two weeks later, while in Vienna, I received the lower centrals which I now exhibit. You observe that it is a very large contour operation, and it is similar to the one I showed him in the mouth of my patient. This proves that gold can be built up by this method, but how it will stand in the mouth, time only can tell. One of the preparations sent by Herbst, involving the mesial, distal, and about one-sixteenth of an inch of the cutting edge of an upper incisor, only required forty minutes' time for the introduction of the gold. This method, therefore, may save a very great deal of time and labor. The principal difficulty in these operations is the making of a proper matrix.

When this has been accomplished the filling is comparatively a simple matter. The matrix is prepared of shellac. (See Figs. 9 and 13.) The proximate walls, as well as the cutting edge, are enclosed by pieces of watch spring fastened into the shellac matrix of the lingual wall. When thus arranged these cavities form a simple cavity with four side walls, into which the gold is easily packed.

*Dr. Brockway*—Do you use single cylinders ?

*Dr. Bodecker*—That varies with the size of the cavity. In small cavities one cylinder is sufficient, but in large ones we may safely employ two or three large cylinders in one layer, and be able to condense them perfectly. If I had the proper instruments here I would extract a few of the fillings from these preparations of Dr. Herbst.

For the present, I do not make contour operations altogether by this method, because I want to be careful, and not experiment on my patients any more than I am justified in doing. The walls and edges of a filling, when made by this method, are better than anything I can do with the mallet. But when the operation is finished with the mallet, I am sure I run no risk, and I save at least half the time I would have used if I had filled the entire cavity by the mallet system.

I desire to mention that Dr. Herbst made most of the preparations exhibited here to-night expressly for this meeting. He sends his cordial greetings, and hopes that every one of you may be benefited by these specimens.

## Figures and Tables

**Fig. 1. f1:**
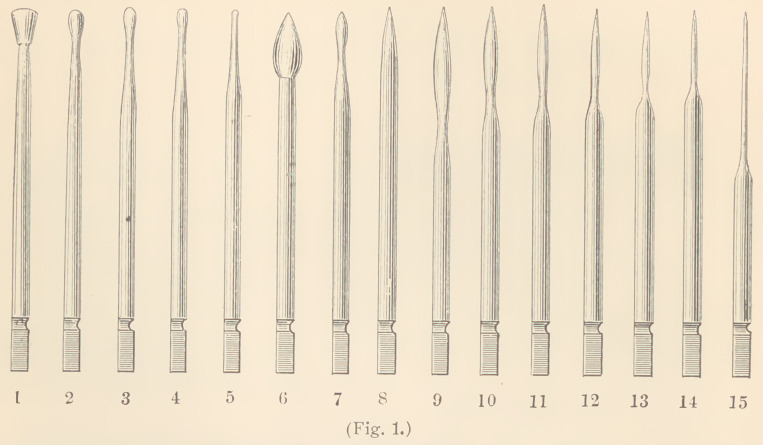


**Fig. 2. f2:**
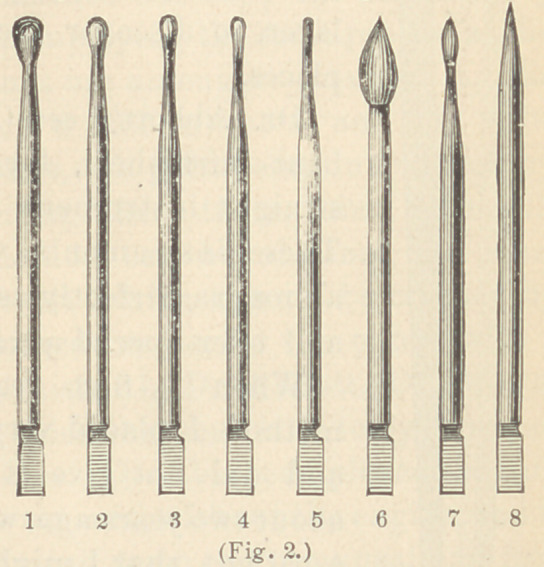


**Fig. 3. f3:**
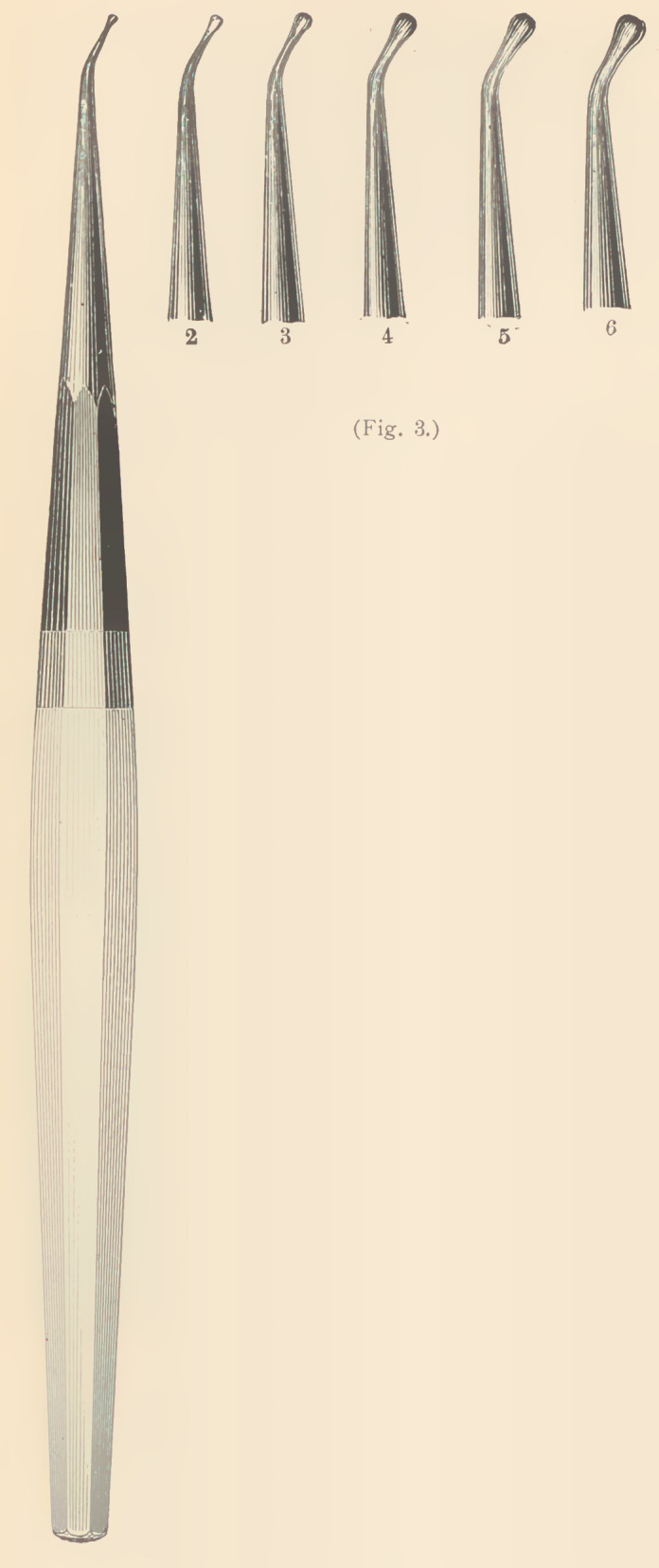


**Fig. 4. f4:**
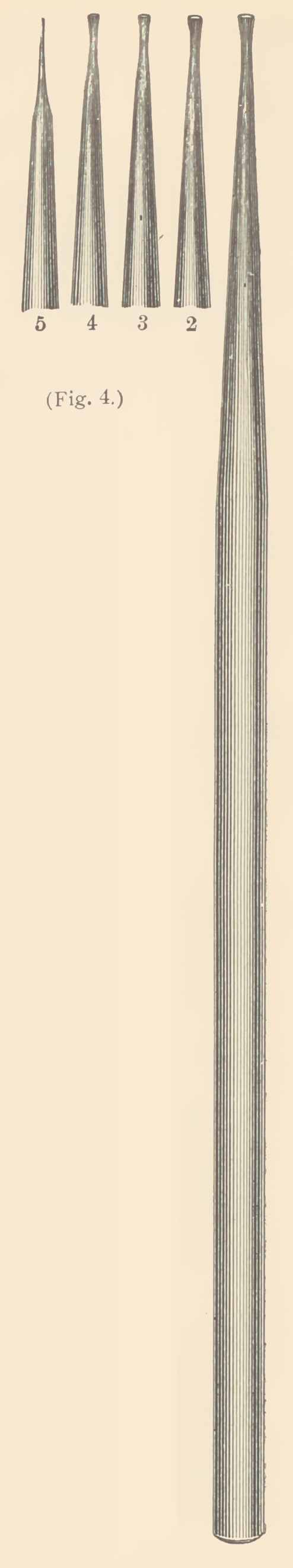


**Fig. 5. f5:**
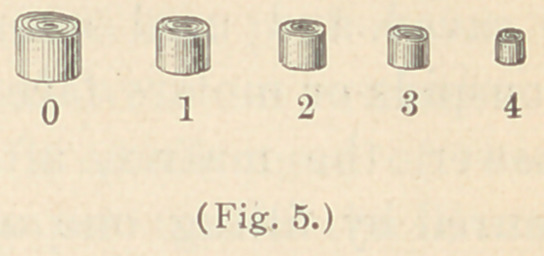


**Fig. 6. f6:**
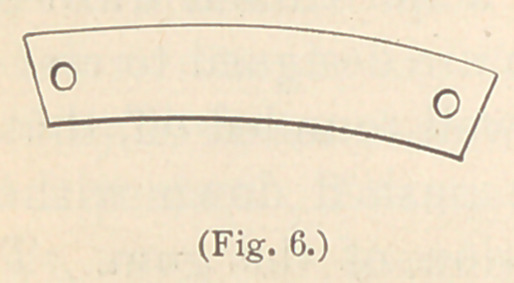


**Fig. 7. f7:**
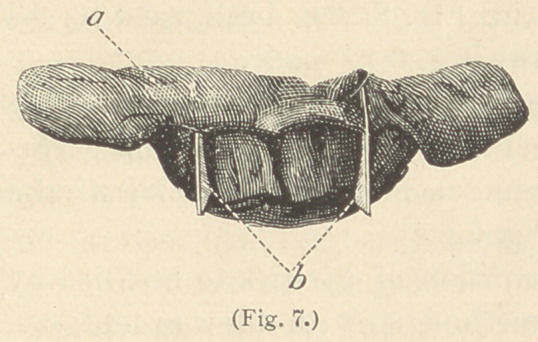


**Fig. 8. f8:**
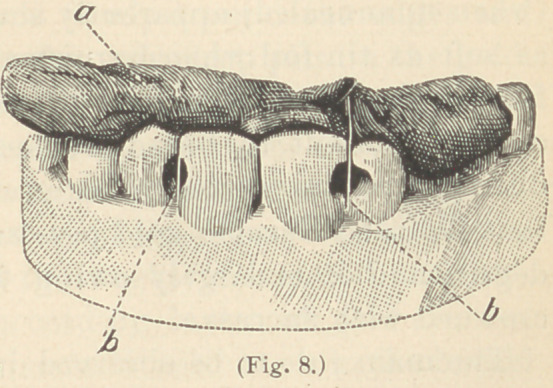


**Fig. 9. f9:**
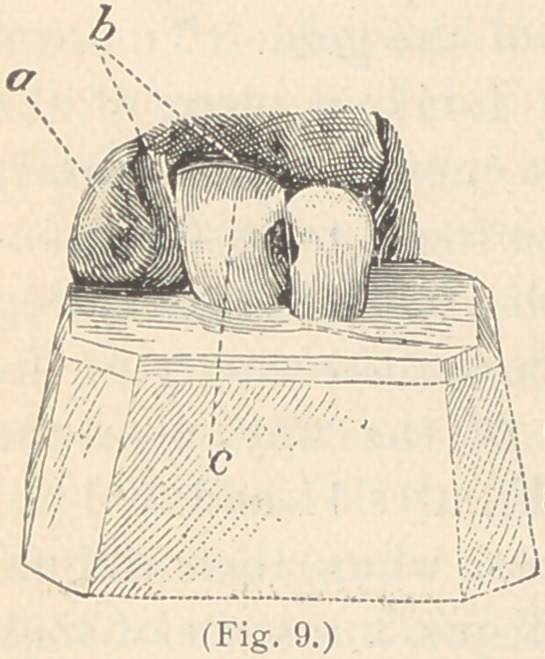


**Fig. 10. f10:**
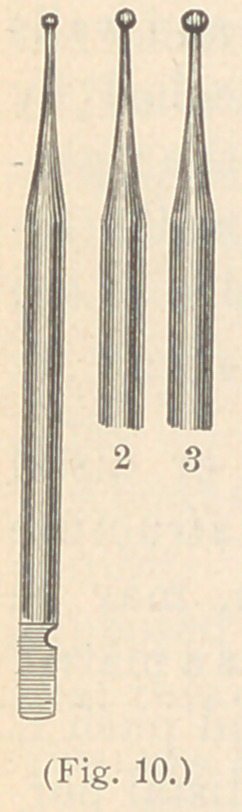


**Fig. 11. f11:**
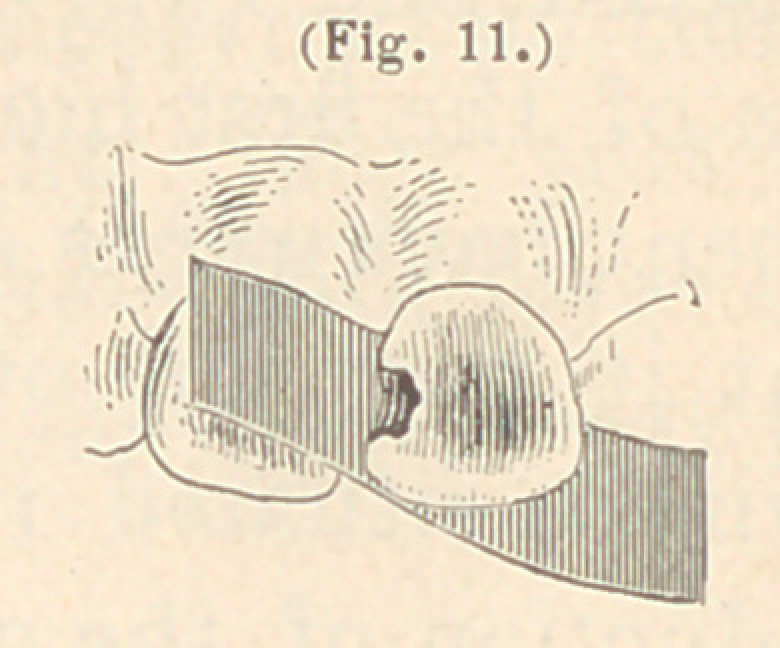


**Fig. 12. f12:**
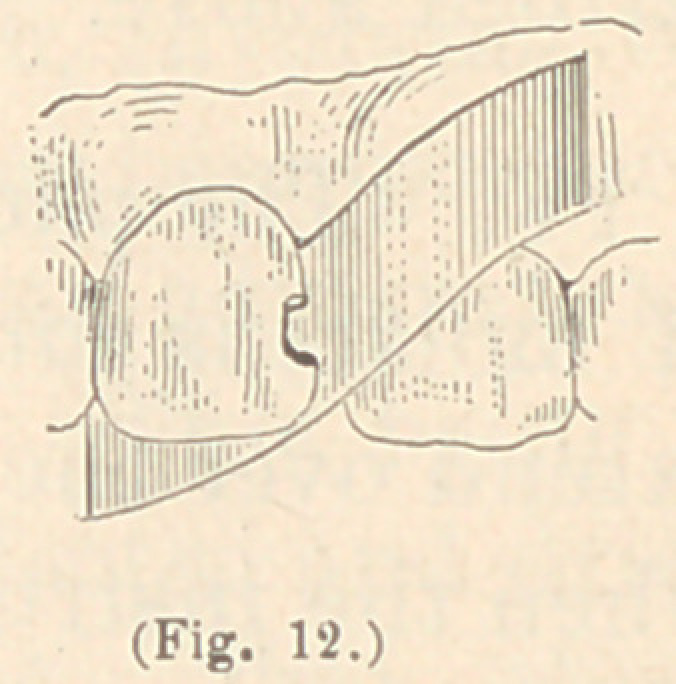


**Fig. 13. f13:**